# A dengue fever predicting model based on Baidu search index data and climate data in South China

**DOI:** 10.1371/journal.pone.0226841

**Published:** 2019-12-30

**Authors:** Dan Liu, Songjing Guo, Mingjun Zou, Cong Chen, Fei Deng, Zhong Xie, Sheng Hu, Liang Wu

**Affiliations:** 1 School of Medicine, Wuhan University of Science and Technology, Wuhan, China; 2 School of Geography and Information Engineering, China University of Geosciences, Wuhan, China; 3 State Key Laboratory of Virology, Wuhan Institute of Virology, Chinese Academy of Sciences, Wuhan, China; 4 National Engineering Research Center for GIS, Wuhan, China; Faculty of Science, Ain Shams University (ASU), EGYPT

## Abstract

With the acceleration of global urbanization and climate change, dengue fever is spreading worldwide. Different levels of dengue fever have also occurred in China, especially in southern China, causing enormous economic losses. Unfortunately, there is no effective treatment for dengue, and the most popular dengue vaccine does not exhibit good curative effects. Therefore, we developed a Generalized Additive Mixed Model (GAMM) that gathered climate factors (mean temperature, relative humidity and precipitation) and Baidu search data during 2011–2015 in Guangzhou city to improve the accuracy of dengue fever prediction. Firstly, the time series dengue fever data were decomposed into seasonal, trend and remainder components by the seasonal-trend decomposition procedure based on loess (STL). Secondly, the time lag of variables was determined in cross-correlation analysis and the order of autocorrelation was estimated using autocorrelation (ACF) and partial autocorrelation functions (PACF). Finally, the GAMM was built and evaluated by comparing it with Generalized Additive Mode (GAM). Experimental results indicated that the GAMM (R^2^: 0.95 and RMSE: 34.1) has a superior prediction capability than GAM (R^2^: 0.86 and RMSE: 121.9). The study could help the government agencies and hospitals respond early to dengue fever outbreak.

## Introduction

Dengue fever (DF), an acute vector-borne disease caused by dengue virus, belongs to the Flaviviridae family, and is transmitted by mosquito vectors [1–3]. People who become infected can develop clinical symptoms with different levels, such as mild fever, headache, muscle and joint pain. In severe cases, bleeding, shock and even death can occur [2–4]. DF spreads widely in tropical and subtropical regions, such as Africa, Americas, Southeast Asia, and the western Pacific [5]. Evidence has shown that nearly half of the world’s population face the threat of DF, and 390 million individuals were infected with dengue per year, of which nearly 96 million have clinical symptoms [6]. In the past 50 years, the incidence of DF has increased 30-fold [7–9]. In the 21st century, DF has transmitted rapidly and has become a serious public health problem.

There were no cases of dengue between 1949 and 1977 in mainland China until the first dengue outbreak occurred in Guangdong province in 1978. Since then, different scales of DF have appeared in Fujian, Guangxi, Zhejiang, and other provinces in China [10, 11]. In recent years, DF has spread further and has also been observed in Henan province of central China. Up to now, there are still no definitive treatment for DF, and popular dengue vaccines have not achieved satisfactory results [12–16]. Therefore, establishing an accurately and early prediction system is an important means for adequate preparedness and response to the outbreak of DF.

Many early warning models were developed in term of different data sources and different methods. The traditional data mainly included dengue cases data, meteorological data, media data (density of mosquito vector) and social factors data. Among them, meteorological data is an important factor for warning models to forecast dengue fever outbreaks [17–19]. Besides, with the development of the Internet in recent years, Internet search data, such as Google, Wikipedia, Yahoo, has been demonstrated as a good complement to traditional monitoring data and can reflect the outbreak of some diseases in some extent. [20–26]. The warning models can be divided into two categories according to analyze the change features of various variables in time latitude: the qualitative warning methods and the quantitative warning methods. The qualitative warning methods estimated disease of development trends and intensity based on the occurrence and development of historical cases. But it relied on the baseline that compared the current number of cases with the historical data in the same period which has a lot of room for improvement in predictive performance [27]. The quantitative warning models predicted future development trend of the disease based on the established mathematical model, such as regression analysis [28–30], time series methods [31–33]. Previous studies have found relationships between DF and meteorological data was non-linear[34]. Nowadays, Generalized Additive Model (GAM) represented by natural cubic splines are the main models used in environmental epidemiology [35–37]. However, GAM is essentially a probabilistic model, explaining how dependent variables depend on independent variables randomly, not showing how dependent variables depend on each other. Besides, GAM requires that each observation is independent, autocorrelation of the cases may cause the problematic estimates of the model.

The aim of this study is to find a better prediction model for estimate and predict the time and scale of the DF. Therefore, we proposed the Generalized Additive Mixed Model (GAMM), which combined the GAM and an autocorrelation term (AR). In this model, we introduced the Dengue Baidu Search Index (DBSI) as variable besides climatic factors (mean temperature, relative humidity, and precipitation) and added the autocorrelation item of DF cases. Firstly, the time series DF data were decomposed into seasonal, trend and remainder components by the seasonal-trend decomposition procedure based on loess (STL). Secondly, the time lag of variables were determined in cross-correlation analysis and the order of autocorrelation was estimated using autocorrelation (ACF) and partial autocorrelation functions (PACF). Finally, the GAMM was built and evaluated by comparing with the GAM. This study can help hospital management to allocate medical resources, and also help us to monitor the abnormal incidence of diseases.

## Materials and methods

### Study setting

Different levels of dengue fever have occurred in China, especially in Guangzhou, Guangdong province, China. Guangzhou, located between 112°57'E and 114°03' E longitude and 22°26'N and 23°56' N latitude, has 12 municipal districts with an area of 7434 km^2^ and a population of about 13 million [38]. It has a maritime subtropical monsoon climate with an annual average temperature of around 22°C, and more rainfall, which is suitable for the growth of mosquito vectors. The above factors increase the risk of dengue transmission.

**Fig 1:**
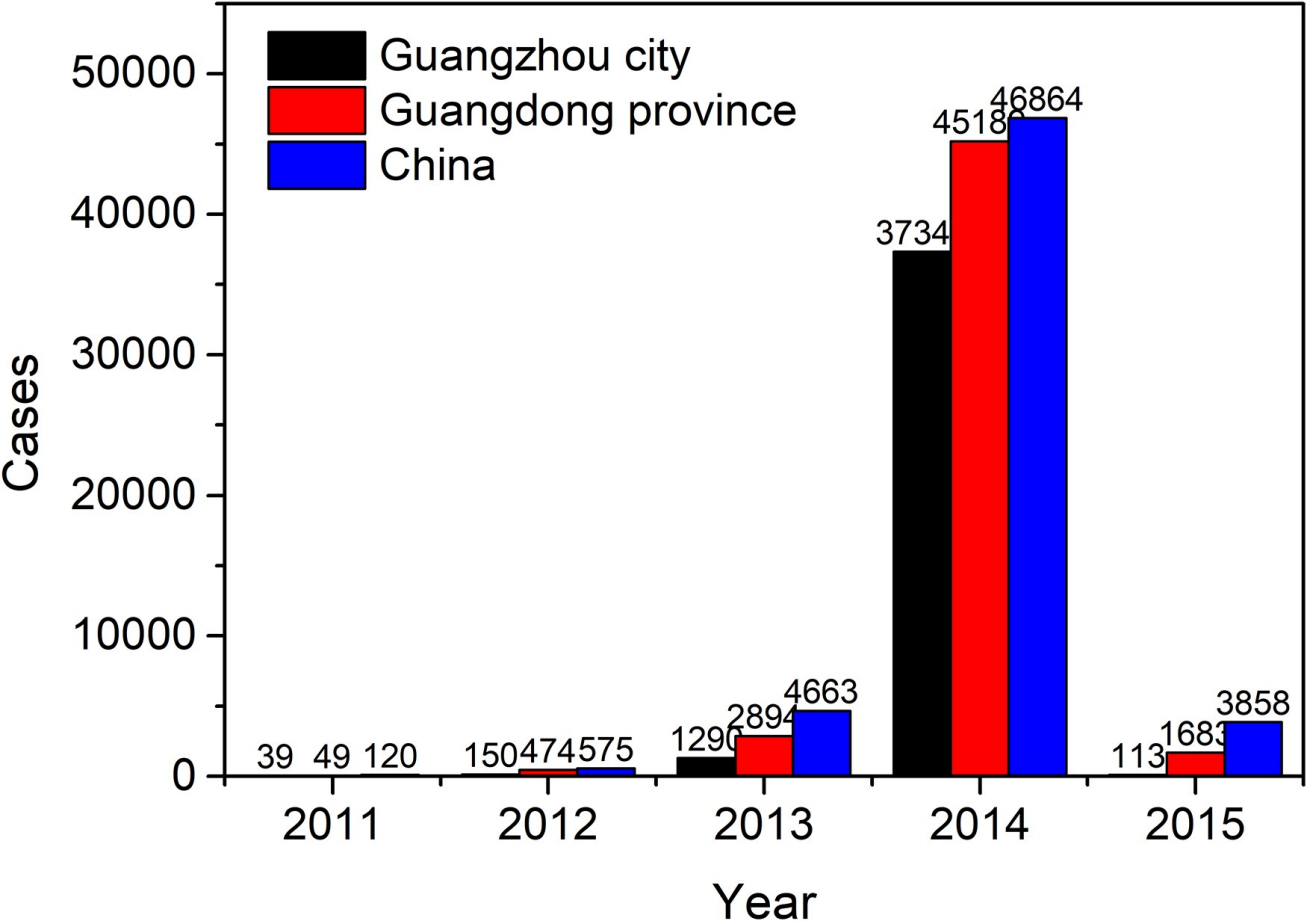
Annual dengue incidence in China, Guangdong, and Guangzhou from 2011 to 2015 respectively.

Guangzhou has always been being a high-risk area for DF in mainland China, and the number of reported dengue cases have ranked first in the country. During 2011–2015, the number of dengue cases in Guangzhou accounted for 77.4% (38932/50289) of the cases in Guangdong Province and 69.4% (38932/56080) of the cases in China during the same period (Fig 1). In particular, in 2014, there were 37,354 dengue cases in Guangzhou city, accounting for 70.57% of the total number of cases in China since 1978 [30]. Hence, Guangzhou is an ideal area to study dengue in China.

### Data sources

#### Dengue cases data

The dengue cases data of Guangzhou from January 2011 to December 2015 in this study were obtained from the Public Health Science Data Center (http://www.phsciencedata.cn/Share/ky_sjml.jsp) whose disease data come from a statutory infectious diseases report database established by Chinese Center for Disease Control and Prevention. The database collected all statutory reported infectious diseases data since 2004. Information of dengue cases included location of the report, sex, age, occupation, the number of the infected in multi-dimension, morbidity, death toll, and mortality. Dengue cases were diagnosed according to the China National Diagnostic Criteria for dengue fever (WS216–2008) enacted by the Chinese Ministry of Health [39].

#### Meteorological data

Meteorological data from January 2011 to December 2015 in Guangzhou were obtained from Guangzhou Meteorological Data Website (http://data.tqyb.com.cn/weather/history_weather_fm.jsp) including the monthly average temperature (°C), monthly mean relative humidity, and precipitation (mm) (S1 Dataset). The climate data used were collected by meteorological station widely distributed in China. The monthly average meteorological data of a city were calculated using the area-weighted average method.

#### The Baidu index data

Baidu is the most popular search engine in China, accounting for more than 80% market share [40]. Therefore, the Baidu search index website (http://ndex.baidu.com/) is used as a data source, which based on the search behavior of users in Baidu. We could obtain the daily counts at the national, provincial, and city level since January 2011. Search data were extracted based on a monthly basis and city level for the study period.

### Statistical analysis

#### Keywords selection and filtering

The network search volume of different keywords could affect the precision of the prediction model. Therefore, the selection of keywords is critical. Unfortunately, there are no clear principles and standards for the selection of keywords [25, 41–43]. Previous studies have mainly used the names of the disease, clinical symptoms, and diagnosis as the main terms to search more related terms. In this study, we obtained the related keywords from a Chinese website (http://tool.chinaz.com/baidu/words.aspx) to minimize the omission of main terms [40]. Typing original terms, we obtained 40 related keywords (S1 Table) suggested by different websites: recommendations of Baidu, portals websites, blogs, and online reports using semantically related analytics. Some recommended keywords may not be closely related to DF occurrence, which could reduce the detective ability of models [28, 44]. Therefore, keywords were filtered as follows: 1) the keywords irrelevant to DF and those with a search volume of zero in the website of the Baidu index were eliminated; and 2) the Spearman’s rank correlation coefficients(*ρ*_1_) between monthly dengue cases and search volumes were calculated during the corresponding period. We excluded the terms with correlation coefficient less than 0.4 and those correlations without statistical significance (*P* > 0.05). 11 keywords were left finally (S2 Table).

The remaining terms were used to combine the DBSI. Weights of terms were defined by the values of the correlation coefficients (ρi). The DBSI was calculated as follow:

$$weight_{i}=\frac{\rho_{i}}{\sum_{i=1}^{n}\rho_{i}}$$ (1)

$$\text{DBSI}=\sum_{i=1}^{n}weight_{i}\times keyword_{i}$$ (2)

Where n is the number of keywords, keyword_*i*_ and weight_i_ represent the *i*th keyword of the search volume in the Baidu index website and the weight of the *i*th keyword, respectively.

#### Seasonal-trend decomposition procedure based on loess (STL)

Local dengue fever of outbreaks are affected by many factors including meteorological factors, medium, and human factors. Therefore, dengue cases data showed obvious volatility and it is difficult to see the seasonal trend from the original time series data. Seasonal-trend decomposition procedure based on loess (STL) can analyze local dengue cases and find whether there were long-term and seasonal trends, which laid the foundation for whether to add the long-term trend and seasonal trend of control in the prediction model. Here, the STL method was used to divide dengue cases data into trend term, seasonal term, and residual term. The expression was as follows:

$$Y_{t}=Trend_{t}+Seasonal_{t}+\text{Re}mainder_{t}$$ (3)

Where *Y*_*t*_ represents the local dengue cases at time t, *Trend*_*t*_ represents the trend term, *Seasonal*_*t*_ represents the seasonal term, *Remainder*_*t*_ represents the residual term, and t is the time in unit of month, t ranges from 1 to N. Long-term trends and seasonal trends of local dengue cases were observed.

#### Establishment of the GAMM

Firstly, the autocorrelation term was determined through the ACF and PACF of dengue cases. Secondly, spearman cross-correlation analysis was carried out to identify the correlation between dengue cases and the average temperature, precipitation, relative humidity, and the DBSI for the lag of 0–6 months. The lag term with the largest Spearman correlation coefficient for each variable was selected. Finally, GAMM was established. Considering the impact of past dengue cases on current dengue cases, the autocorrelation term was also added to improve the accuracy of the predicting model. Because dengue was a small probability event related to the entire population and satisfied the Poisson distribution, the study used the quasi-Poisson model to allow for excessive dispersion of dengue cases data. The smooth natural cubic spline functions on these risk factors were used to reflect the non-linear association between dengue cases and each dependent variable. In order to evaluate the quality of the proposed model, we made comparisons between the GAMM and the GAM without adding the autocorrelation term. The basic form of the models to be constructed were as follows:

$$\text{ln}[E(\mu_{t})]=\beta_{0}+s(T_{t-a},df)+s(H_{t-b},df)+s(P_{t-c},df)+s(DBSI_{t-d},df)+year+month$$ (4)

$$\text{ln}[E(\mu_{t})]=\beta_{0}+s(T_{t-a},df)+s(H_{t-b},df)+s(P_{t-c},df)+s(DBSI_{t-d},df)+year+month+\alpha_{0}$$ (5)

$$\alpha_{0}=\sum_{j=1}^{p}C_{j}\left((\text{ln}(\mu_{t-j}^{*})-\sum_{i=1}^{m}f(X_{t-ji})\right)$$ (6)

Formula (4) and (5) are expressions of the GAM and the GAMM respectively. μ_t_ is the number of dengue cases in month t, E(*μ*_*t*_*)* is the expected value of the t-month of dengue cases, log(*) is the link function of model, and β_*0*_ is a constant term. Additionally, *s(T*_*t-a*_, *df)* is the natural cubic spline function of the average temperature in the previous a month with the corresponding *df*, *s(H*_*t-b*_, *df)* is the natural cubic spline function of the relative humidity in the previous b month with the corresponding *df*, *s(P*_*t-c*_, *df)* is the natural cubic spline function of the precipitation, in the previous c month with corresponding *df*, *s(DBSI*_*t-d*_, *df)* is the natural cubic spline function of the DBSI in the previous d month with corresponding *df*. Year controls long-term trends, and month controls seasonal trends. α_0_ is an autocorrelation. y* = max(y, τ), *τ* = 0.5, *τ* is to prevent *y* from being 0 and negative.

In the models, the *df* directly or indirectly affect the fitting effect of the model, and the choice of *df* values were essential. In this study, the *df* of independent variables were determined by the local minimum principle of Akaike Information Criterion(AIC)[45]. Autocorrelation term (*P* value) was determined by ACF and PACF for dengue cases. In addition, the model had to further control the volatility of long-term trends and seasonal trends so that the average temperature, relative humidity, 8precipitation, and the DBSI could better estimate the number of dengue cases.

In this study, we divided the disease data into two parts: The model was developed on the data from January 2011 to June 2015 and validated using the remaining data. For the GAM and GAMM, R^2^ was applied to evaluate the goodness of model fitting. Larger R^2^ values were associated with stronger explanatory ability of the model. Furthermore, in order to fully evaluate the model, the ACF and PACF of the residual were used to test whether the residual of the model was independent. Moreover, we also analyzed the relationships between the Pearson residual and the time to test independence hypothesis of the model. If the Pearson residual of the model did not change with time, the model conformed to the independence hypothesis.

Finally, the root mean square error (RMSE) was devoted to evaluate the quality of the model, which tested the consistency between local dengue cases and predicted cases of the two models. The RMSE reflects the error of circumstances between the actual and predicted values. Smaller RMSE was associated with better prediction ability.

$$\text{RMSE}=\sqrt{\frac{\sum_{t=1}^{n}{\left({\overset{\_}{y}}_{t}-y_{t}\right)}^{2}}{n}}$$ (7)

Where ${\bar{y}}_{t}$ is the actual of local dengue case data at time t, *y*_*t*_ is the predicted value of dengue case data in the model at time t, and n is the size of samples for prediction.

All the analyses were performed in R version 3.4.0. Results with *P* values of less than 0.05 were considered statistically significant in all statistical tests. The model was built and analyzed using the “mgcv” package. All relevant data are within the paper and Supporting Information files. The code needed to reproduce the results presented here are available on github at https://github.com/guoandwu08180914/hellodata123.git.

## Results

### Factors influencing dengue cases

**Fig 2:**
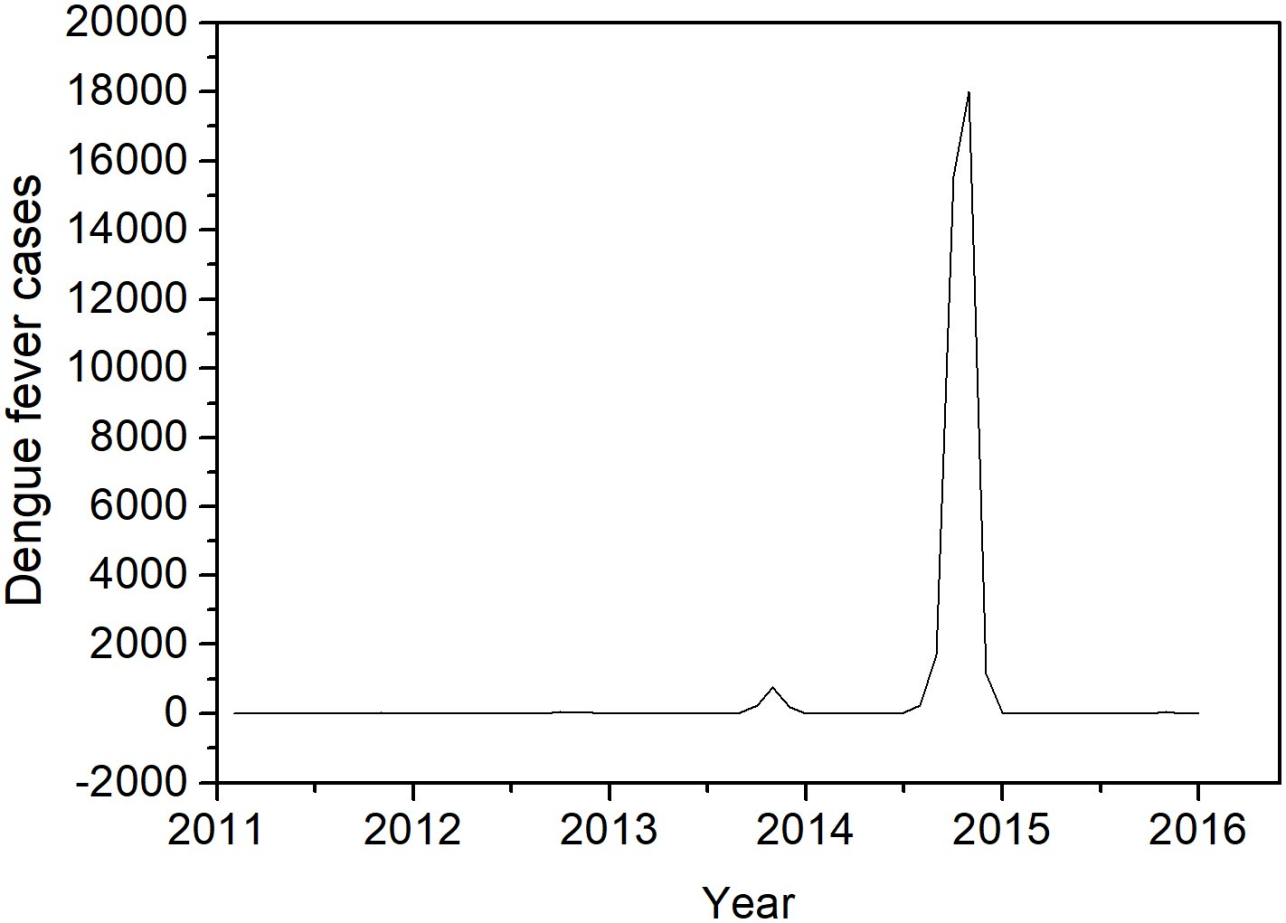
Temporal distribution of dengue fever cases in Guangzhou, 2011–2015.

During the period from 2011 to 2015, dengue fever cases of Guangzhou have indicted different degrees every year, mainly occurred from August to November, late summer and autumn, with a large outbreak in 2014 (Fig 2). The monthly average temperature, relative humidity, and precipitation all shows seasonal fluctuations and reaches the peak at April to September, March to July, May to October, respectively. Moreover, relative humidity and precipitation increased year by year, whereas average temperature do not change substantially. The time series of the monthly DBSI is significantly similar to the time series of monthly dengue cases, which also shows two peaks in 2013 and 2014. The peak in 2014 is particularly prominent, indicating that the DBSI could reflects changes in the dengue epidemic (S1 Fig).

**Fig 3:**
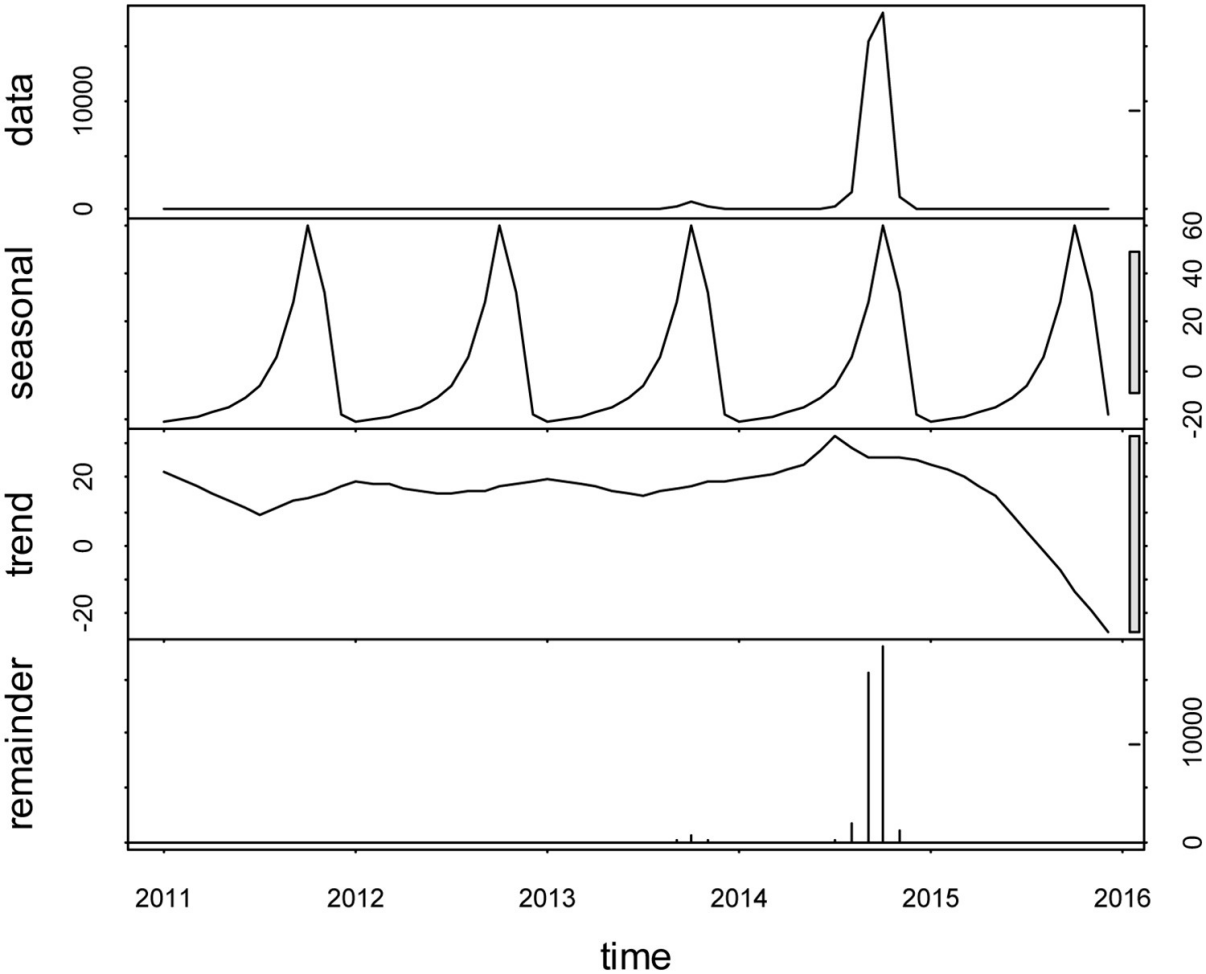
The decomposition plot of local dengue cases in the study areas from January 2011 to December 2015.

The decomposition result showed that DF in Guangzhou city had an increased treed from 2011 to 2014, and then mildly decrease in 2015. Besides, DF also had a seasonal distribution, which were prone to have more case from August to November every year. Therefore, long-term and seasonal effects on local dengue cases should be considered when building the prediction model (Fig 3).

**Fig 4:**
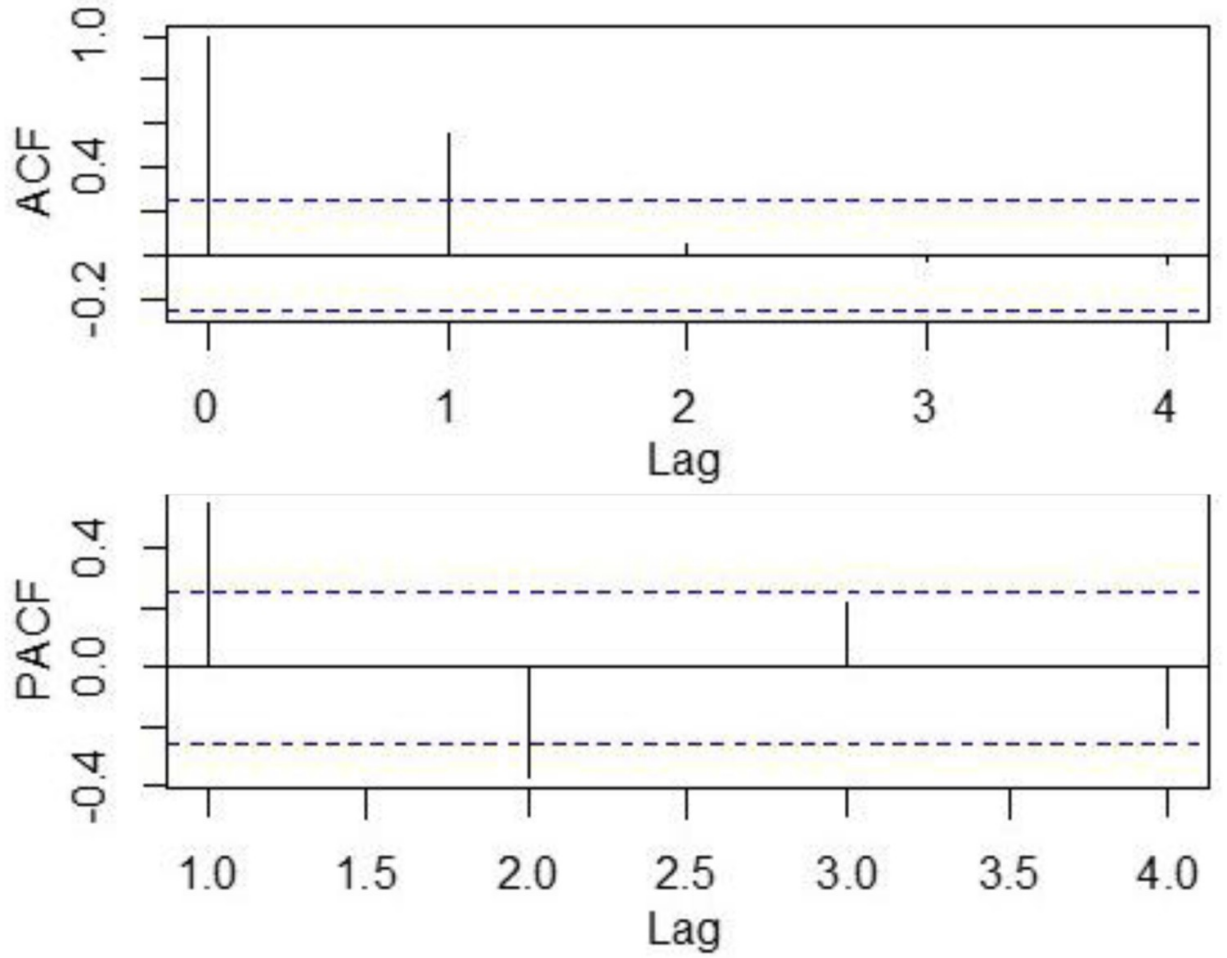
Auto-correlation and partial auto-correlation plots of dengue cases, 2011–2015.

The auto-correlation analysis showed that the autocorrelation coefficient with a lag of 1 and the partial auto-correlation coefficient cutoff at lag 2, indicating that the number of dengue cases at lags 1 months affects the number of dengue cases in the current month (Fig 4). Therefore, the autocorrelation item of dengue cases must be considered when building the model. According to the results of the autocorrelation analysis, the *P* value of the autocorrelation item was set to 1.

Spearman cross-correlation analysis of dengue fever cases with monthly mean temperature, monthly relative humidity, monthly cumulative rainfall, and DBSI with a lag time of 0–6 months showed that the DF had the strongest cross-correlation coefficients with lag of 2 months for the average temperature, lag of 3 months for relative humidity, lag of 3 months for precipitation, and lag of 0 month for DBSI, their Spearman cross-correlation coefficients are 0.718, 0.605, 0.692, and 0.734, respectively (Table 1).

**Table 1:** Cross-correlation analysis between dengue cases and average temperature, relative humidity, precipitation, DBSI.

	Temperature	Humidity	Precipitation	DBSI
**Lag0**	0.404*	-0.073	0.055	0.734*
**Lag1**	0.618*	0.143	0.238	0.672*
**Lag2**	0.718*	0.456*	0.497*	0.536*
**Lag3**	0.619*	0.605*	0.692*	0.416*
**Lag4**	0.356*	0.600*	0.594*	0.270
**Lag5**	0.022	0.444*	0.318	0.135
**Lag6**	-0.361	0.225	-0.022	0.024

Notes: Each positive answer equals 1 point,

*p<0.05.

### Measures of the ability of model fitting and forecasting

**Fig 5:**
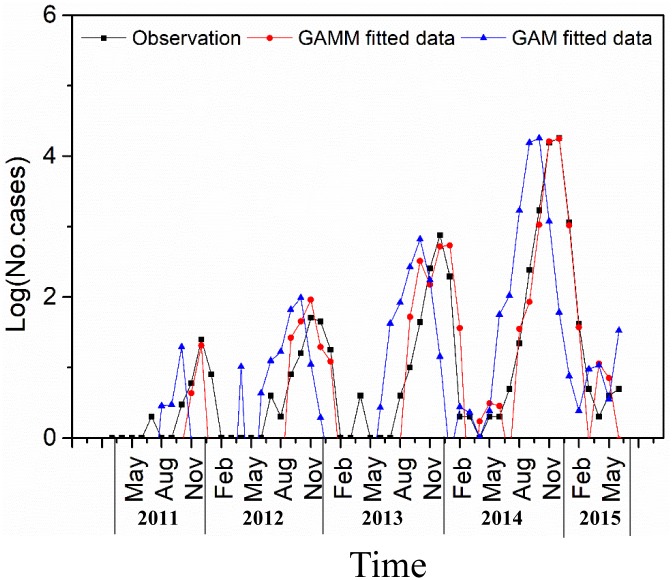
Monthly observed DF cases and fitted local DF cases using two different models from January 2011 to June 2015.

**Fig 6:**
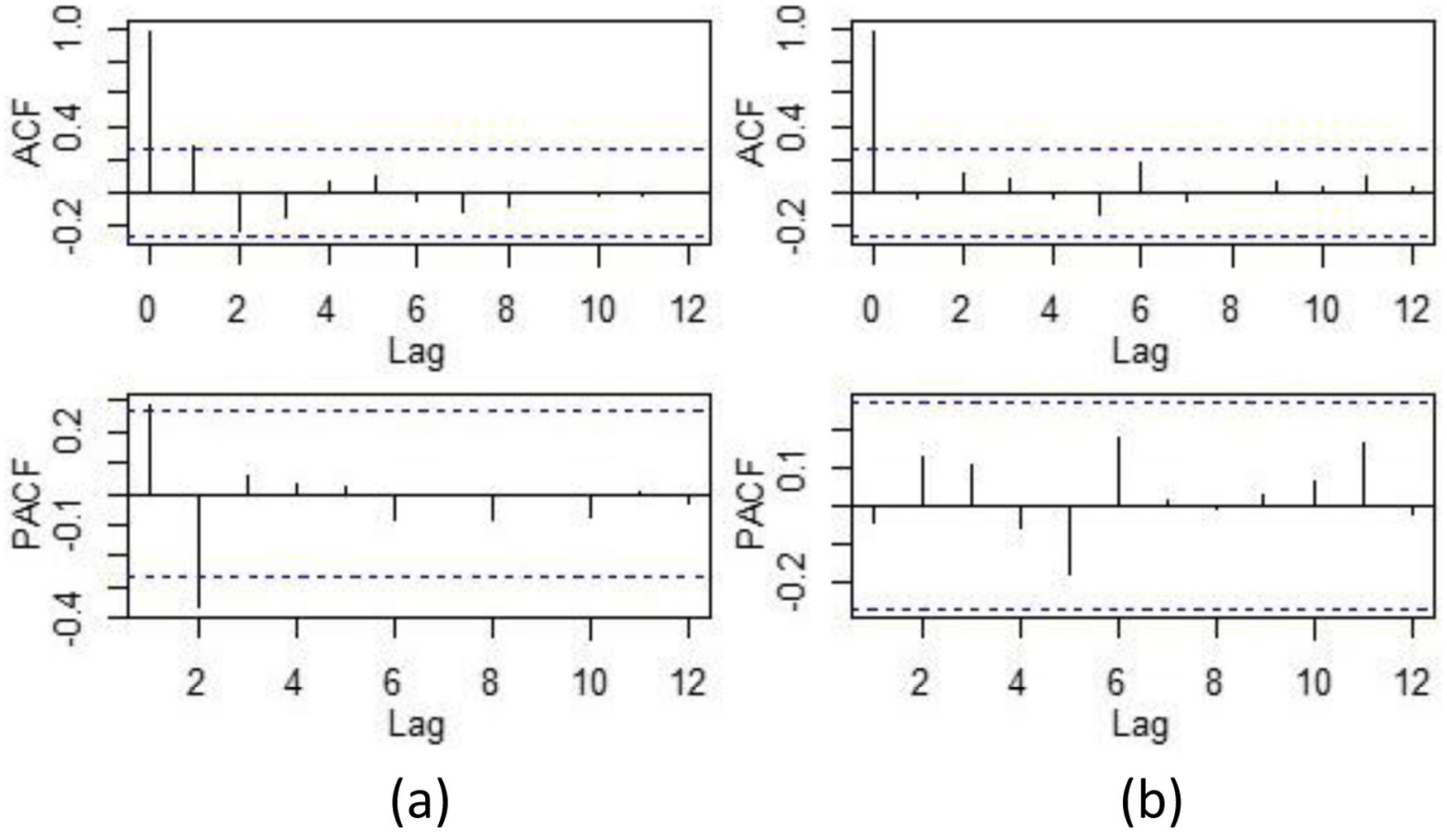
Auto-correlation and partial auto-correlation of residuals. (a) ACF/PACF plot of the Pearson residual of the GAM. (b) ACF/PACF plot of the Pearson residual of the GAMM.

The R^2^ of GAM was 0.86 and the R^2^ of GAMM was 0.95. The fitting results exhibited that GAMM has a better fitting performance compared with GAM (Fig 5). The residual test (Fig 6) by ACF and PACF showed residuals of GAM have obvious auto-correlation. However, residual in the GAMM were not correlated, which indicated that the information of these variables was extracted sufficiently.

**Fig 7:**
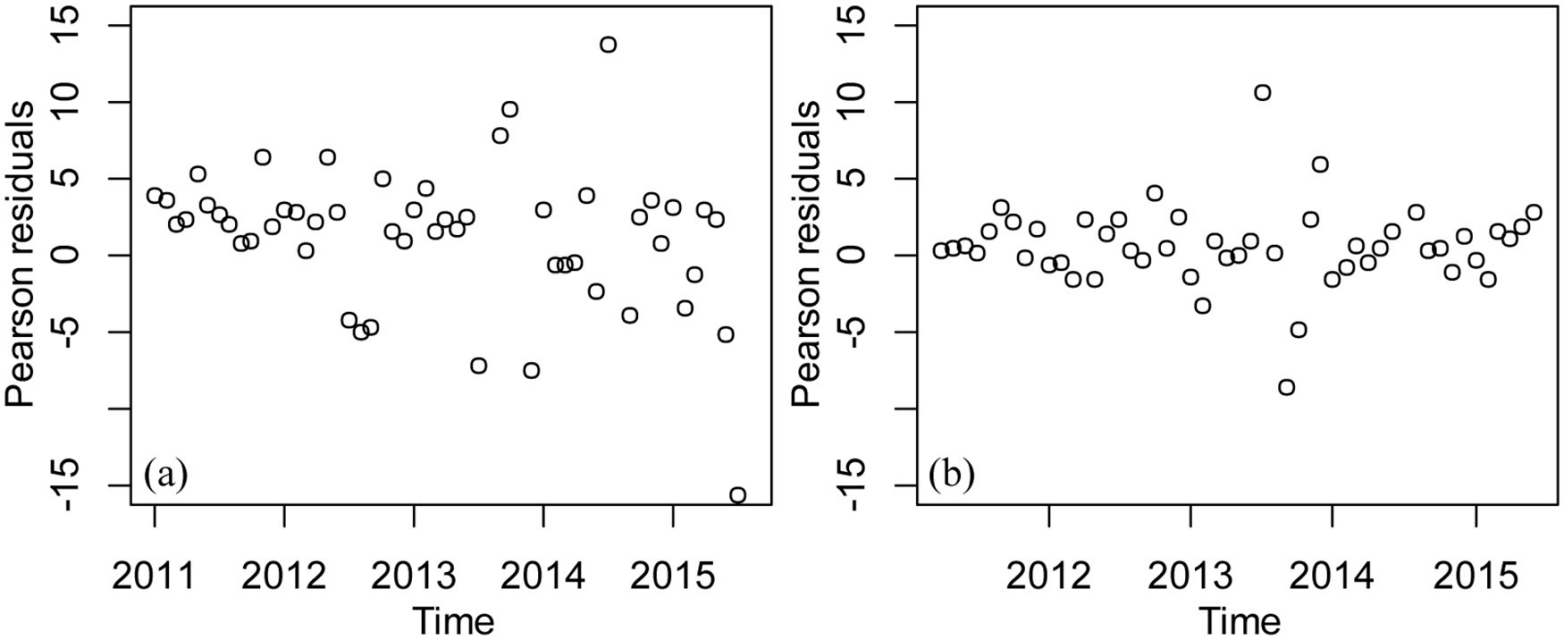
Scatter plot of residuals for predicted values using two different models and dates. (a) Scatter plot of residual for predicted values and dates in the GAM. (b) Scatter plot of residual for predicted values and dates in the GAMM.

**Fig 8:**
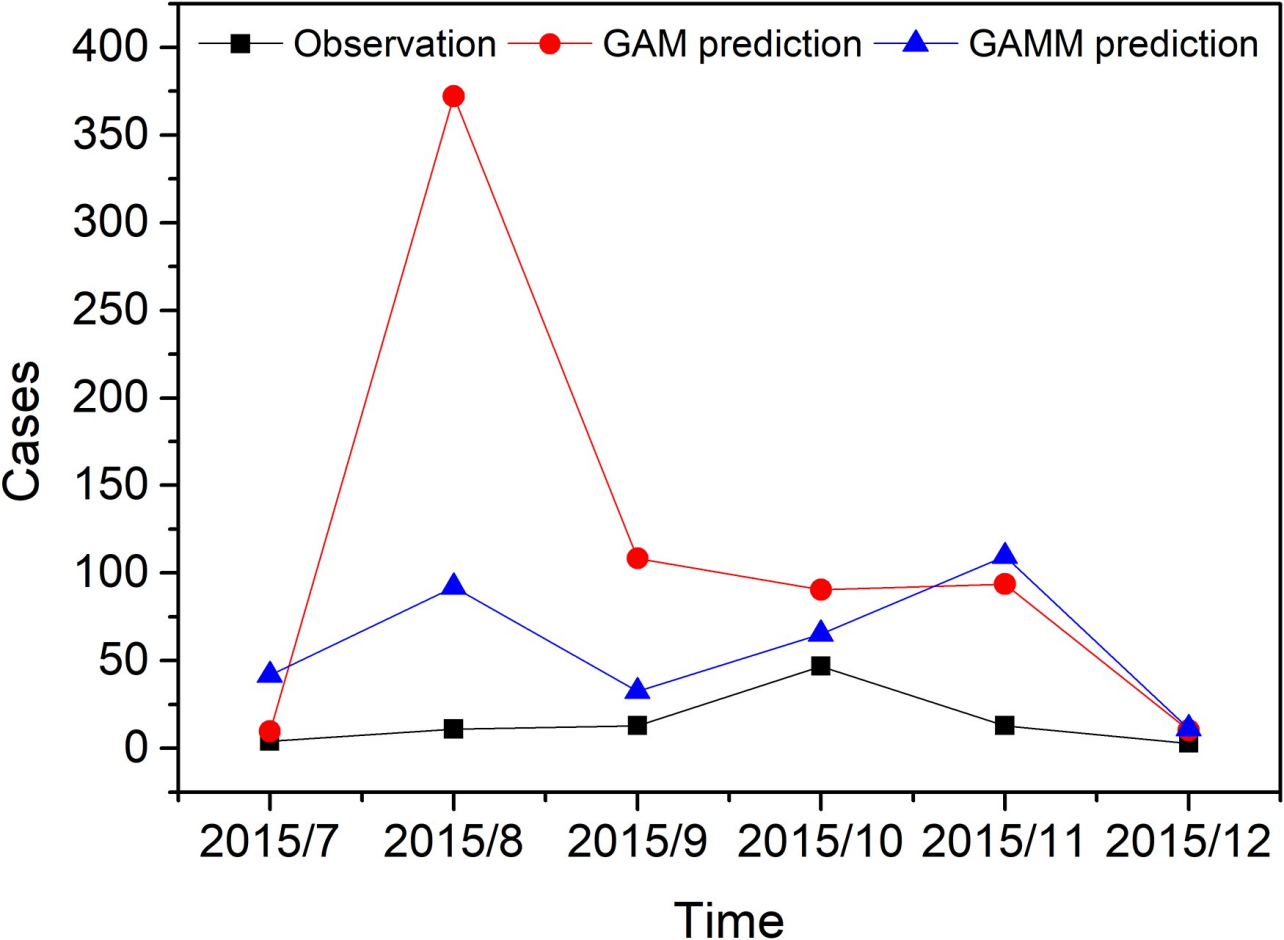
Observations and model predictions of DF case using two different models from July 2015 to December 2015.

Besides, the Pearson residual of the GAM model has significant fluctuations over time, violating the model assumptions of GAM (Fig 7). The forecast result showed that the GAMM (RMSE: 121.9) gives a better prediction of DF cases than the GAM (RMSE: 34.1) (Fig 8).

## Discussion

Since the beginning of the 21st century, DF has become one of the most serious infectious diseases worldwide and has seriously affected public health in many countries. From January 2011 to December 2015, there were 38,277 cases of dengue fever in Guangzhou city, accounting for 76% of dengue cases in Guangdong Province during the same period. In 2014, Guangzhou city experienced the largest outbreak of dengue fever in history, during two months (September and October), the number of cases of dengue fever exceeded 30,000, which was higher than the number of cases in other countries and regions, leading to panic in Guangzhou residents. Given that there are currently no specific drugs for dengue treatment and no reliable vaccine for prevention, the World Health Organization (WHO) believes that the establishment of early warning system is a crucial means to deal with the outbreak of dengue fever.

Accurate and timely forecasts of dengue incidence is essential in China. Government and hospitals can timely grasp the time and scale of DF outbreak to take preventive and control measures that reduce the number of dengue cases. In this study, we introduced meteorological factors (mean temperature, relative humidity, precipitation) and DBSI into the GAMM model. The autocorrelation item of dengue cases was also considered in the prediction model. The results indicated that the proposed model could effectively monitor dengue fever cases in Guangzhou city for a short period.

DF is transmitted form of human-mosquito-human. At present, there is no effective vaccine against dengue in China. The control of mosquitoes is the most effective way to prevent DF in endemic countries. For example, *wolbachia* [46, 47], spray pesticides, and genetic modification [48] have achieved good effects. However, the establishment of an early DF warning system that enhance the predictability of dengue outbreaks is still a key step in dengue control. Various countries, including Nepal[49] Thailand [50], Singapore [51], Indonesia [52–54], Venezuela [55], and Trinidad [56], have conducted numerous studies on DF, which reported that meteorological factors play a crucial role in dengue transmission by direct or indirect effects on mosquito vectors[57, 58]. The impact of climate on dengue fever is not immediate but accumulates over time. Study found that the mosquito-borne mating rates were highest at 20°C and 25°C [59]. In this study, dengue cases of Guangzhou city are sharp increase in August to September, lag after two months (during June to July), the average annual temperature of Guangzhou city of 20°C to 25°C, which is consistent with previous studies. Notably, during this period, the growth of *Aedes* can be reduced by spraying with syrup or eliminating water accumulation. Our study also indicated that lag of 3 months for relative humidity had a large impact on the number of dengue. The possible reasons are that eggs and adult mosquitoes are affected by relative humidity, and the number of mosquito ovulations and adult mosquitoes are reduced in dry areas [60]. Besides, we also found that precipitation in the previous 3 months was positively correlated with dengue case in current month that agreement with previous studies [61], highlighting the importance of precipitation in the spread of dengue fever. Heavy precipitation can flush away the egg, larvae and pupae of *Aedes* mosquitoes in the short term, prevent the growth of mosquito vectors. In addition, precipitation shorten the time that people are exposed to mosquitoes and the rate of mosquito bites, leading to reduce the risk of DF infection in a certain extent. However, precipitation can also provide an excellent breeding condition for the growth of mosquito larvae, increasing the number of mosquitoes in the long run [62, 63].

When users search information by the Internet, the frequency of the specific phrases, time and place and other relevant information are preserved by web search engine, which reflect the behavior tendency of the Internet users. Internet search data has comprehensive, massive and real-time characteristics, which can better reflect the tendency and timeliness of people’s social behavior. With the rapid development of Internet technology, more and more people use Internet search for health information, which provides data basis for disease monitoring model. At present, many scholars have used Google, Yahoo, Wiki, Twitter, and other web searches to conduct research to predict the prevalence of seasonal diseases and have made certain achievements [22, 24, 26, 64]. According to the 39th Statistical Report on Internet Development, the total number of Internet users in China was 73.1 million by 2016, accounting for about 53.2% of the national population. Most of them use the Baidu engine[65]. Therefore, we added the DBSI to the model and found that there was a strong correlation (coefficient of association 0.73424) between current month DBSI and dengue cases in Guangzhou city, which suggests that dengue patients are usually aware of their condition and will conduct relevant Internet searches current month before see a doctor. Study proves that DBSI can provide a supplement f traditional disease surveillance.

Moreover, we validated our model by comparing the actual DF values and the predicted values of the GAM and GAMM in the last 6 months of 2015, we further discovered that the GAMM with autocorrelation item had better performance in the dynamic monitoring of dengue cases in Guangzhou city. The GAM only explained the effects of the climate factors and the DBSI on the change of DF, not included an unpredictable long-term trend of DF, whereas the GAMM took into account the maximum past information that affected the current dengue cases, which was more robust for adjusting the long-term trend. Therefore, the GAMM was a better choice for prediction models.

Although the study achieved a good result, there were some limitations to the model. Firstly, the amount of sample data was small. In this study, we only used data from January 2011 to December 2015. The monthly accuracy data for dengue cases in 1990–2015 could be obtained from the Public Health Science Data Center website, however, we could only obtain the Baidu search data after January 2011. Therefore, this study contains 5 years of experimental data at most, and this may have affected the robustness of the model. In future studies, weekly/day data can be considered to replace monthly data. Secondly, Baidu search data account for the largest market for Internet data in China and have become the largest data source for tracking Chinese search behavior. However, these data do not represent all Internet data. Moreover, the Baidu search index provides a dimensionless data, and the official website does not give a specific calculation method, indicating that these data only partly reflect the trends in dengue cases. Besides, dengue Baidu search behaviors are easily affected by the news media and government, resulting in uncertainties in such search data. There is no specific way to overcome this problem currently. In further studies, we will combine the commonly used Weibo data in China with the Baidu data to build models and evaluate media-driven interests or other events that change search behavior. Finally, other risk indicator data that may be associated with dengue fever does not examine, such as population data, socio-economic, urbanization status, mosquito control measures, and animal herd immunity.

## Conclusions

In the 21st century, dengue fever has transmitted rapidly and has become a serious public health problem. Early warning system is great important to the treatment and prevention of DF. Therefore, in this study, we introduced the Dengue Baidu Search Index (DBSI) as variable besides climatic factors (mean temperature, relative humidity, and precipitation) and added the autocorrelation item of DF cases to establish the GAMM. Experimental results indicated that the GAMM has a superior prediction capability. The study could offer the assist with public health interventions to prevent and control the dengue outbreak.

## Supporting information

S1 Dataset Meteorological data. (XLSX)

S1 Fig Temporal distribution of climate variables and Baidu search variable from July 2015 to December 2015. (TIF)

S1 Table Search keywords from Baidu index website in this study. (XLSX)

S2 Table Dengue related Baidu search terms that were finally selected. (XLSX)
